# Improving newborn care practices through home visits: lessons from Malawi, Nepal, Bangladesh, and Uganda

**DOI:** 10.3402/gha.v8.23963

**Published:** 2015-03-31

**Authors:** Deborah Sitrin, Tanya Guenther, Peter Waiswa, Sarah Namutamba, Gertrude Namazzi, Srijana Sharma, KC Ashish, Sayed Rubayet, Subrata Bhadra, Reuben Ligowe, Emmanuel Chimbalanga, Elizabeth Sewell, Kate Kerber, Allisyn Moran

**Affiliations:** 1 Save the Children, Washington, DC, USA; 2 School of Public Health, Makerere University, Kampala, Uganda; 3 Save the Children, Kathmandu, Nepal; 4 UNICEF, Kathmandu, Nepal; 5 Save the Children, Dhaka, Bangladesh; 6 National Institute of Population Research and Training, Ministry of Health and Family Welfare, Dhaka, Bangladesh; 7 Save the Children, Lilongwe, Malawi; 8 Department of Neonatology, Children's National Medical Center, Washington, DC, USA; 9 Save the Children, Cape Town, South Africa

**Keywords:** newborn, neonatal, postnatal, home visit, community-based, community health worker

## Abstract

**Background:**

Nearly all newborn deaths occur in low- or middle-income countries. Many of these deaths could be prevented through promotion and provision of newborn care practices such as thermal care, early and exclusive breastfeeding, and hygienic cord care. Home visit programmes promoting these practices were piloted in Malawi, Nepal, Bangladesh, and Uganda.

**Objective:**

This study assessed changes in selected newborn care practices over time in pilot programme areas in four countries and evaluated whether women who received home visits during pregnancy were more likely to report use of three key practices.

**Design:**

Using data from cross-sectional surveys of women with live births at baseline and endline, the Pearson chi-squared test was used to assess changes over time. Generalised linear models were used to assess the relationship between the main independent variable – home visit from a community health worker (CHW) during pregnancy (0, 1–2, 3+) – and use of selected practices while controlling for antenatal care, place of delivery, and maternal age and education.

**Results:**

There were statistically significant improvements in practices, except applying nothing to the cord in Malawi and early initiation of breastfeeding in Bangladesh. In Malawi, Nepal, and Bangladesh, women who were visited by a CHW three or more times during pregnancy were more likely to report use of selected practices. Women who delivered in a facility were also more likely to report use of selected practices in Malawi, Nepal, and Uganda; association with place of birth was not examined in Bangladesh because only women who delivered outside a facility were asked about these practices.

**Conclusion:**

Home visits can play a role in improving practices in different settings. Multiple interactions are needed, so programmes need to investigate the most appropriate and efficient ways to reach families and promote newborn care practices. Meanwhile, programmes must take advantage of increasing facility delivery rates to ensure that all babies benefit from these practices.

Nearly all deaths in newborns (99%) occur in low- or middle-income countries (1). While there has been global progress towards United Nation's (UN) Millennium Development Goal 4 to reduce mortality in children younger than age 5, only 23 of 75 priority countries are on track to meet their goals (2). Reducing the number of deaths during the newborn period is increasingly important to reaching those targets, because neonatal mortality now accounts for 43% of under-5 mortality (3).

In 2011, the World Health Organization (WHO) led a review of evidence of the impact of various reproductive, maternal, newborn, and child health interventions and recommended essential interventions for scale-up. Several interventions for newborns were recommended for delivery at community level, including promotion and provision of thermal care, early and exclusive breastfeeding, and hygienic cord care (4). Table 1 outlines ways in which these practices benefit the newborn.

**Table 1:** Benefits of newborn care practices promoted at community level

Practices	Specific behaviours	Benefits to newborn
Breastfeeding	Initiation of breastfeeding within 1 hour after birth	Reduced risk of death, particularly from infectious disease due to protective factors in colostrum and breast milk and protection
	Exclusive breastfeeding for 6 months	from ingestion of contaminants found in water, other fluids, and
	Continued breastfeeding with safe complementary foods after 6 months	food with exclusive breastfeeding (5) Reduced risk of morbidity, including gastro-intestinal infections and respiratory infections (6)
Cord care	Cord cut with sterile blade and cord care with sterile tie/clamp	Reduced risk of death and morbidity due to tetanus and sepsis, omphalitis, cord infection (7–10)
	Nothing harmful applied to cord (topical antibiotic/antiseptic may be recommended in some settings)	
Thermal care	Immediate drying	Reduced risk of hypothermia and sequelae including (11–16)
	Delayed bathing	∘Death
	Wrapping in clean cloth and/or skin-to-skin care	∘Infection ∘Central nervous system depression leading to bradycardia, apnoea, and poor feeding ∘Coagulation defects, haemorrhage

One method for promoting these practices in the community is through home visits, and there have been a number of studies demonstrating that home visits by trained community health workers (CHWs) can change newborn care practices (17–20). Countries with high neonatal mortality have increasingly adopted postnatal home visit policies (2) in response to the 2009 release of the WHO and United Nations Children's Fund Joint Statement recommending home visits during pregnancy and soon after delivery to reduce newborn deaths (21). Save the Children's Saving Newborn Lives (SNL) programme supported ministries of health and partners in Malawi, Nepal, Bangladesh, and Uganda to develop and test newborn programmes that use existing health system structures to deliver services for newborns, including home visits by community-based employees or volunteers (collectively called CHWs) affiliated with the Ministry of Health or other government agencies (22–25). Pilot areas were selected for implementation and learning in all countries. In pilot areas of Malawi, Nepal, and Bangladesh existing CHWs were given in-service training and support to do home-based maternal and newborn care and counselling, and were encouraged to integrate these home visits into their work. In Uganda, Ministry of Health guidelines were used to select new volunteer CHWs (called Village Health Teams or VHTs) because the VHT policy was in place, but the programme had not yet been scaled up. Selected CHWs were trained in a maternal and newborn care package.

The purpose of this analysis was to evaluate population-level changes in selected newborn care practices over time in pilot areas in four countries with different health system contexts and to learn whether women who received home visits from CHWs were more likely to report use of these practices.

## Methods

### Settings and programme descriptions

Programmes in all four countries included a package of community-based care for women and newborns delivered through a series of home visits during pregnancy and after birth. To deliver these services, programmes used existing (or sanctioned) community-based health workers or volunteers and support systems, with SNL and other partners providing training in maternal and newborn care; supplies required to deliver maternal and newborn services; and support for supervision, monitoring, and community engagement. In addition to home visits, programmes also included facility strengthening, mainly through training, supplies, and supervision, although the extent of this varied across countries. Likewise, community engagement efforts differed: CHWs in Malawi organised ‘core groups’ for planning and decision making; CHWs in Nepal worked with existing mothers’ groups; community leaders in Bangladesh attended orientation sessions; and research staff in Uganda sensitised district staff, traditional birth attendants, health workers, and private providers about the interventions and the study.

Approximately 600 CHWs in Malawi, 850 in Nepal, 100 in Bangladesh, and 61 in Uganda were trained on maternal and newborn care in pilot intervention areas. Duration of trainings ranged from 5 to 9 days. CHWs were instructed to make 2–4 home visits during pregnancy and 2–4 home visits after the birth (the number of visits varied across countries). Existing supervision systems were strengthened to support and encourage visits. The type of compensation that CHWs received was different and included salaries, stipends, and small travel allowances. CHWs in Nepal received compensation tied to the number of visits (see Table 2). As a result of these and other factors, coverage of visits varied widely across countries (26).

**Table 2:** Community worker cadre, characteristics, training, and incentives

	Bangladesh		Malawi	Nepal	Uganda
Cadre name	• Family Welfare Assistant (FWA) • Health Assistant (HA) – Females only • Community Nutrition Promoter (CNP)		Health Surveillance Assistant (HSA)	Female Community Health Volunteer (FCHV)	Village Health Team (VHT)
Characteristics	FWA/HA:	CNP:			
• Gender	• Female	• Female	• Mostly male	• Female	• Mixed
• CHW: pop. ratio	• 1:6,000–7,000	• 1:1,250	• 1:1,000–2,000	• 1:400^a^	• 1:1,000
• Education level	• Secondary education	• Primary (not strict)	• Secondary	• Literate, primary preferred	• Literate
• Employment status	• Govt-salaried employee	• Volunteer (stipend)	• Govt-salaried employee	• Volunteer	• Volunteer (travel allowance)
• Recruitment	• From communities	• From communities	• Centrally recruited	• From communities	• From communities
• Pre-service training	• 21 days (FWA) 6 weeks (HA)	• 24-day training	• 12-week training	• 18-day training	• None
Training in maternal newborn community package	5 days		9 days (+6 days community mobilisation)	6 days	6 days
Paid incentives based on number of visits	No		No	Yes	No

a Catchment area population size varies in Nepal depending on terrain; 1:400 ratio is applicable to Terai region such as Bardiya.

Home visits included promotion of optimal maternal and newborn care and use of routine facility services; counselling on danger signs and care-seeking for mother and baby; screening for newborn danger signs by a physical assessment including checking the baby's temperature, breathing, and weight; and referral to a health facility when needed. Although coverage of home visits varied across countries, most mothers reported that key functions were likely to be performed when the visits occurred (26). The package in Nepal included curative care limited to CHW administration of one dose of oral antibiotics to newborns with possible severe bacterial infection. They then referred the sick newborn and caregiver to a health post to complete treatment with injectable antibiotics. In Nepal, CHWs were also expected to attend home deliveries and conduct resuscitation, if needed.

To help promote healthy newborn care practices, CHWs were equipped with counselling cards to use during home visits. Table 3 shows the visit schedule and the messages CHWs were trained to give women and families related to the newborn care practices evaluated in this study.

**Table 3:** Home visit schedule and messages given during home visits on newborn care practices

		Malawi	Nepal	Bangladesh	Uganda^a^

		Chitipa, Dowa, Thyolo districts	Bardiya district	Faridpur district	Iganga & Mayuge districts
# Pregnancy visits		3 (1st, 2nd and 3rd trimester)	4 (4th, 6th, 8th, 9th months)	2 (2nd and 3rd trimester)	2 (early as possible and 3rd trimester)
# Postnatal visits		2–3 (day 1 – home births only, 3, 8)	4 (day 1, 3, 7, 29)	3 (day 1, 2–3, 4–7)	2 (day 1–3, 5–7)
Early initiation of breastfeeding	Messages	Initiate breastfeeding within 30 min after delivery depending on feeding option chosen by mother	Initiate breastfeeding within 1 hour after birth	Initiate breastfeeding immediately and no later than 1 hour after birth	Put baby on the breast within 1 hour of birth
	When delivered	2nd and 3rd pregnancy visit 1st postnatal visit (home births)	3rd and 4th pregnancy visit 1st postnatal visit (home births)	All pregnancy visits	1st postnatal visit
Delayed bathing	Messages	Delay bathing the baby for 24 hours after birth	Newborn should not be bathed for at least 24 hours after birth	No bath within 3 days of birth	Delay bathing until after the 1st day
	When delivered	2nd and 3rd pregnancy visit 1st postnatal visit (home births)	3rd and 4th pregnancy visit 1st postnatal visit (home births)	All pregnancy visits	1st postnatal visit
Skin-to-skin care	Messages	Place the baby on the mother's abdomen for skin-to-skin contact to promote bonding	Place the newborn close to mother and make him/her warm	Place naked baby on mother's bare chest or abdomen within 24 hours of birth Mother and infant should be covered with a clean, dry cloth	Dry the baby. Then put the naked baby between your breasts. Cover him or her lightly with a dry cloth and keep in this position as much as possible during day and night.^b^
	When delivered	2nd and 3rd pregnancy visit 1st postnatal visit (home births)	3rd and 4th pregnancy visit 1st postnatal visit	All pregnancy visits	2nd postnatal visit
Cord care	Messages	Avoid applying traditional herbs or substances on the umbilical cord	Keep the cord dry and clean Do not put oil or anything onto the cord	Cord should be kept clean and dry after cutting Apply nothing to cord stump If cord is very soiled it may be cleansed gently with boiled water	Do not put anything on the cord, keep it clean and dry Let the cord stump dry and fall off on its own
	When delivered	All 3 postnatal visits	3rd and 4th pregnancy visit 1st postnatal visit (home births)	All pregnancy visits	1st postnatal visit

a Counselling cards indicate that counselling on these behaviours should occur during the first postnatal visits. According to the study PIs, the CHWs were encouraged to deliver messages during all pregnancy and postnatal visits during training and supervision.

b In the VHT manual, skin-to-skin care recommended for low birthweight babies. Study PI says VHTs in study area trained to recommend skin-to-skin care for all newborns.

The evaluation designs differed. In Malawi and Nepal, a before–after design was used with no comparison area. In Bangladesh, a before–after design was used with a comparison area. In Uganda, a cluster randomised controlled trial was done. In Malawi implementation areas were parts of three districts (Chitipa, Dowa, Thyolo) with a population of 711,000. In Nepal, implementation was in Bardiya district in the Terai area with a population of 460,000. In Bangladesh, implementation was in four rural unions in Madhukhaliupazila (sub-district) in Faridpur district with a population of 98,000, and an additional four unions in Nagarkandaupazila in Faridpur district were used as comparison areas. In Uganda, 65 villages in Iganga and Mayuge districts with a total population of 70,000 were randomly allocated – 33 to the intervention arm and 32 to the control arm. Additional details on programme design and evaluation have been published previously (26, 27).

### Data collection

Household surveys were conducted before implementation (baseline) and after at least 1 year of implementation (endline). Women with a live birth in the previous 12 months (past 3–12 months at endline in Bangladesh) were interviewed. Start of full implementation to endline data collection was 12 months in Malawi (June 2010–June 2011), 17 months in Nepal (January 2010–June 2011), 14 months in Bangladesh (April 2009–June 2010), and 25 months in Uganda (August 2009–September 2011). The data collection methods have also been described previously (26, 27). In Bangladesh and Uganda, data were also collected in comparison areas.

Caesarean deliveries were excluded from analysis for Malawi, Bangladesh, and Uganda since certain newborn care practices may be delayed after such births and the procedure may affect mothers’ recall. Caesarean delivery rates were 6% or lower in Malawi and Uganda during the whole study period and in intervention and comparison areas. In Bangladesh, caesarean delivery rates were 7.6% in intervention areas and 6.3% in comparison areas at baseline, but jumped to 15.1% in intervention areas at endline while only increasing to 8.1% in comparison areas. The doubling of caesarean delivery rates may reflect the doubling of facility delivery rates in intervention areas in Bangladesh. It was not possible to exclude caesarean deliveries from the Nepal dataset as the mode of birth was not recorded; the rate of caesarean delivery is less than 5% in the sub-region where the study was implemented, according to the 2011 Demographic and Health Survey (28).

### Variables

#### Changes in newborn care practices over time and cross-country comparisons

To examine changes in newborn care practices after roll-out of community-based programmes, we selected key breastfeeding, thermal protection, and cord care practices over which mothers are most likely to have some control, regardless of where the birth takes place, and could thus be influenced by counselling and messages. In addition, practices were selected based on comparability over time and across countries and the mother's ability to respond. Based on these criteria, the following behaviours were included: early initiation of breastfeeding (≤1 hour after birth); delayed bathing (≥6 hours after birth); skin-to-skin contact after birth (data on skin-to-skin only collected at endline); and nothing being applied to the cord. Cord-cutting practices were collected, but could not be included in analysis due to large numbers of women who did not know what was used to cut the cord. WHO recommendations on postnatal care (29) advise delayed bathing for 24 hours after birth, but if not possible due to cultural reasons, delaying at least 6 hours. We used 6 hours for analysis as per these recommendations.

In Nepal only women who gave birth at home were asked about substances applied to the cord. Since few women interviewed in Nepal delivered at home, the percentage reporting nothing being applied to the cord may not be representative and, therefore, was not included in analysis for this country. In Bangladesh, all women were asked about breastfeeding initiation, but only those who gave birth outside a facility were asked about the other practices. However, the majority of women gave birth outside a facility (74%), so analysis for Bangladesh included only non-facility births. In Uganda, the question on timing of first bath was added to the questionnaire during baseline survey implementation, leading to high numbers of missing values, so baseline data are not reported. Also, interviewers in Uganda recorded a categorical response on the timing of breastfeeding initiation at baseline, while the time in hours or days was recorded at endline. This change made the endline questionnaire comparable to other countries, but may have affected internal comparability over time. For the purpose of evaluating changes over time, missing values were excluded from the denominator to avoid skewing the absolute percentage change over time.

#### Pregnancy home visits and newborn care practices

A composite indicator of newborn care practices was created to determine if women who received home visits from CHWs during pregnancy were more likely to report use of healthy newborn care practices at endline. The composite indicator and main outcome variable for this analysis includes early initiation of breastfeeding bathing delayed at least 6 hours, and skin-to-skin contact. Applying nothing to the cord was not included in the composite indicator because we wanted to include only those indicators for which we had endline data from all countries. As explained above, we could not report endline data from Nepal on cord care because only women who gave birth at home were asked the appropriate questions and the numbers were small. For the purpose of creating a composite, missing values for any of the practices were treated as if the woman reported that the practice was not used or responded ‘I don't know’.

The main independent variable was home visits during pregnancy, which was defined categorically (0, 1–2, and 3+ visits) to learn if receiving more visits had a greater association with use of key newborn care practices. Analyses controlled for receipt of antenatal care (0 versus at least 1 visit), place of birth (facility versus non-facility), maternal age (<20, 20–29, 30 + ), and maternal education (any versus none). For Bangladesh, the analysis excluded women who gave birth at a facility since data were not collected from these women due to skip patterns in the questionnaire, as previously mentioned.

### Statistical analyses

The Pearson chi-squared test, corrected for the survey design with second-order correction of Rao and Scott (30), was used to assess changes in practices over time. Generalised linear models (GLM) were used to assess the relationship between the main independent variable, home visit from a CHW during pregnancy, and the composite outcome indicator. Relative risks (RR) and 95% confidence intervals (CI) were obtained. Clustering was controlled for in all analyses; appropriate standard error estimates were produced for the GLM using the Taylor linearisation method (31). Stata version 11 was used (32).

### Ethical considerations

All programmes were implementing national policy through routine systems. Ethical clearance was obtained from the National Health Sciences Research Committee in Malawi, the Bangladesh Medical and Research Council, Makerere University School of Public Health in Uganda, and the Uganda National Council of Science and Technology. As per approved applications to the institutional review boards, women gave verbal consent to participate in the survey in Bangladesh and Malawi and written consent in Uganda. In Nepal, data collection was completed as part of routine programme activities. Relevant district authorities granted permission, and all respondents provided verbal consent upon being informed of the purpose of data collection.

## Results

### Changes in newborn care practices

Table 4 shows newborn care practices at baseline and endline in each country, and the absolute percentage change over time. Missing values were less than 3% for each practice, unless otherwise noted in the table footnotes.

**Table 4:** Newborn care practices at baseline and endline and absolute percent change over time

		Malawi intervention			Nepal intervention			Bangladesh^a^ intervention			Bangladesh^a^ comparison			Uganda intervention			Uganda comparison		
					
		Pre (%)	Post (%)	% change	Pre (%)	Post (%)	% change	Pre (%)	Post (%)	% change	Pre (%)	Post (%)	% change	Pre (%)	Post (%)	% change	Pre (%)	Post (%)	% change
Early initiation of breastfeeding	Yes	74	95	21*	64	90	26*	68	75	8	69	80	11*	54	86	32*	47	85	38*
	No/DK	26	5		36	10		32	25		31	20		46	14		53	15	
Delayed bathing ≥6 hours	Yes	64	91	27*	78	95	17*	67	87	20*	63	73	10*	NC	90		NC	84	
	No/DK	36	9		22	5		33	13		37	27			10			16	
Skin-to-skin care	Yes	NC	68		NC	76		NC	80		NC	73		NC	82		NC	77	
	No/DK		32			24			20			27			18			23	
Nothing applied to cord	Yes	72	71	−1	NA	NA		48	79	31*	57	63	6	44	64	20*	58	52	−6
	No/DK	28	29					52	21		43	37		56	36		42	48	

a Non-facility births only.

* Statistically significant change from baseline to endline at *p*<0.05.

NC=not collected; DK=don't know; NA=not available (questions on substances applied to the cord were only asked for non-facility births in Nepal).

Missing values <3% except: 16.7% missing early initiation of breastfeeding at baseline in Malawi, 9.2% missing delayed bathing at baseline in Malawi, 10.8% missing delayed bathing at endline in Malawi, 6.2% missing delayed bathing at baseline in Nepal. Missing values excluded from denominator to calculate absolute percentage change. Shaded portion distinguishes data from comparison areas for the 2 countries that collected data from non-intervention areas.

In intervention areas there were statistically significant improvements in all practices, except applying nothing to the cord in Malawi and early initiation of breastfeeding in Bangladesh. Statistically significant changes in intervention areas ranged from 17% (delayed bathing in Nepal) up to 32% (early initiation of breastfeeding in Uganda).

In Bangladesh and Uganda, where data were collected from comparison areas, most practices increased in both the intervention and comparison areas. However, in Bangladesh endline proportions were higher in the intervention than the comparison areas for delayed bathing, skin-to-skin contact, and nothing applied to the cord (*p*<0.05). The increase in early breastfeeding was borderline significant in intervention areas (*p*=0.06), while it was statistically significant in comparison areas (*p*=0.01), but the endline proportions were not statistically significantly different between intervention and comparison areas. In Uganda, endline proportions of delayed bathing and nothing applied to the cord were higher in intervention areas than comparison areas (*p*<0.05). The increase in early breastfeeding was greater in comparison areas, but there was no difference between areas at endline.

### Number of practices applied

The number of women who reported use of 0, 1, 2, or all 3 of the newborn care practices included in the composite indicator at endline is shown in Table 5, as well as the average number of practices used. In all countries, half or more of interviewed women reported that all three practices were done. Very few women reported none of these practices being applied.

**Table 5:** Number of practices used at endline, among the three practices included in composite indicator

	Malawi (N=844)		Nepal (N=615)		Bangladesh^a^ (N=293)		Uganda (N=869)	
			
No. of practices	n	%	n	%	n	%	n	%
0	6	1	8	1	3	1	9	1
1	49	6	33	5	33	11	51	6
2	367	44	155	25	93	32	252	29
3	422	50	419	68	164	56	557	64
Mean	2.43		2.60		2.43		2.56	

a Non-facility births only.

### Association between pregnancy home visits and newborn care practices

Table 6 shows the distribution of independent variables for women interviewed in intervention areas at endline and asked about practices included in the composite outcome indicator (early initiation of breastfeeding, delayed bathing, and skin-to-skin contact). Nearly all women in Nepal and Bangladesh reported at least one home visit during pregnancy from a CHW and the majority reported 3 or more visits. In Malawi, 62.1% of women reported no home visit while only 7.6% reported 3 or more. In Uganda, about a third of women fell into each category – no visit, 1–2 visits, and 3 or more visits. Nearly all women in Malawi and Uganda received antenatal care (95.3 and 95.7%), but fewer did in Nepal (65.5%) and Bangladesh (42.3%). Facility birth rates were highest in Malawi (92.1%), followed by Nepal (81.5%), then Uganda (77.3%). These rates were higher than 2010/2011 national averages (28, 33, 34). Facility delivery rates were much lower in Bangladesh (26.4%); only data from non-facility births are included in the analysis.

**Table 6:** Distribution of independent variables for women asked about the three practices included in composite indicator at endline

	Malawi (N=844)		Nepal (N=615)		Bangladesh^a^ (N=293)		Uganda (N=869)	
			
	n	%	n	%	n	%	n	%
Pregnancy home visit								
0	524	62	18	3	26	9	288	33
1 or 2	248	29	89	14	78	27	300	35
3+	64	8	508	83	189	65	279	32
Missing	8	1	0	0	0	0	2	<1
Antenatal care								
None	32	4	212	34	169	58	36	4
At least one visit	804	95	403	66	124	42	832	96
Missing	8	1	0	0	0	0	1	<1
Place of delivery								
Home	54	6	114	19			197	23
Facility	777	92	501	81			672	77
Missing	15	2	0	0			0	0
Maternal age (years)								
<20	121	14	96	16	41	14	109	13
20–29	473	56	453	74	186	63	485	56
30+	230	27	66	11	66	23	275	32
Missing	20	2	0	0	0	0	0	0
Maternal education								
None	103	12	258	42	54	18	97	11
Any	736	87	357	58	239	82	772	89
Missing	5	1	0	0	0	0	0	0

a Non-facility births only.

Table 7 shows the unadjusted and adjusted RR that all three practices were used. In Malawi, Nepal, and Bangladesh women visited once or twice during pregnancy were more likely to report that all three practices were used, but the difference was not statistically significant. In Malawi, women were 34% more likely to report that their newborns were breastfed early, bathed ≥6 hours after birth, and given skin-to-skin contact if visited at least three times during pregnancy, controlling for other independent variables (*p*<0.05). In Bangladesh, where only non-facility births were included in the analysis, women were 88% more likely to report that their newborns received all three recommended practices if visited at least three times during pregnancy (*p*<0.05). The same trend was seen in Nepal, but very few women received no visits during pregnancy and there was no statistically difference between women who received at least three visits and those that received none. In Uganda women who received pregnancy home visits were not more likely to report that their newborns received all three practices compared to women that did not receive a home visit. However, when we did separate analysis for each behaviour, delayed bathing was statistically significantly associated with three or more pregnancy home visits in Uganda.

**Table 7:** Generalised linear models for four countries with a composite indicator of early initiation of breastfeeding, delayed bathing, and skin-to-skin contact at endline as the dependent variable

	Malawi				Bangladesh^a^				Nepal				Uganda			
			
	n	Crude RR	Adjusted (95% CI)	*p*	n	Crude RR	Adjusted (95% CI)	*p*	n	Crude RR	Adjusted (95% CI)	*p*	n	Crude RR	Adjusted (95% CI)	*p*
Pregnancy home visits																
None (Ref)	489	1.00	1.00		26	1.00	1.00		18	1.00	1.00		287	1.00	1.00	
1 or 2	235	1.07	1.07 (0.91–1.27)	0.410	78	1.30	1.36 (0.77–2.39)	0.278	89	1.52	1.37 (0.89–2.12)	0.143	299	0.99	1.01 (0.90–1.14)	0.819
3+	62	1.36	1.34 (1.11–1.62)	0.003	189	1.83	1.88 (1.06–3.35)	0.032	508	1.55	1.45 (0.95–2.20)	0.082	277	1.00	1.01 (0.89–1.15)	0.840
ANC^b^ visit																
None (Ref)	29	1.00	1.00		169				212	1.00	1.00		36	1.00	1.00	
At least one	757	1.06	0.97 (0.65–1.45)	0.893	124	1.12	1.14 (0.90–1.44)	0.265	403	1.05	1.00 (0.88–1.13)	0.977	827	1.38	1.29 (0.90–1.85)	0.153
Place of delivery																
Non-facility (Ref)	46	1.00	1.00		0	1.00	1.00		114	1.00	1.00		196	1.00	1.00	
Facility	740	2.20	2.25 (1.28–3.93)	0.005	293	–	–	–	501	2.10	2.11 (1.56–2.84)	0.000	667	1.48	1.47 (1.30–1.66)	0.000
Maternal age																
<20 (Ref)	114	1.00	1.00		41	1.00	1.00		96	1.00	1.00		109	1.00	1.00	
20–29	453	1.23	1.22 (0.99–1.51)	0.056	186	1.21	1.25 (0.82–1.89)	0.287	453	1.00	1.00 (0.87–1.16)	0.962	480	1.15	1.16 (1.01–1.33)	0.041
30+	219	1.29	1.30 (1.02–1.66)	0.034	66	1.06	1.03 (0.68–1.56)	0.886	66	0.90	0.94 (0.77–1.14)	0.516	274	1.04	1.11 (0.93–1.32)	0.252
Maternal education																
None (Ref)	90	1.00	1.00		54	1.00	1.00		258	1.00	1.00		95	1.00	1.00	
Any	696	1.11	1.09 (0.84–1.41)	0.534	239	1.10	1.00 (0.77–1.31)	0.996	357	1.03	0.94 (0.83–1.06)	0.312	768	0.99	0.98 (0.85–1.12)	0.711
Total	786				293				615				863			

a Non-facility births only.

b Antenatal care (at LEAST one visit with a skilled provider).

<10% missing one or more independent variable and excluded from model (approximately 7% missing values in Malawi, 1% in Uganda and 0% in Nepal and Bangladesh).

Mothers were more likely to report use of all three practices if the birth happened at a facility. In Malawi older mothers were also more likely to have reported use of all three practices. In Uganda women aged 20–29 years were more likely to have reported these practices, but not mothers over 30 years.

## Discussion

This multi-country study provides important information on the extent to which community-based packages can be expected to influence care for newborns. The packages examined in this analysis all included home visits by CHWs to promote and support evidence-based newborn care practices, with varying emphasis on other inputs such as facility strengthening.

There were sharp increases in most selected newborn care practices over a short period of time, in all countries and in both intervention and comparison areas. Changes cannot be completely attributed to the community-based interventions, and there are a number of other factors that may have contributed, including the rapid rise in facility delivery rates. Whether or not the birth took place at a facility appears to be the most important contributor to use of the selected practices. Yet use of all three practices (early initiation of breastfeeding, delayed bathing, and skin-to-skin contact) was still moderate in Malawi, Nepal, and Uganda (49, 68, and 64%, respectively) where facility birth rates were high. Thus increasing the promotion and use of these practices is still needed at health facilities.

As noted, changes in behaviours over time need to be interpreted with caution, given the dynamic environment and changes in measurement from baseline to endline. However, it is interesting to note the lack of change in dry cord care in Malawi, where mothers who deliver at a facility may have little control over this practice. Further, the CHW training manual included messages about appropriate cord care under items to discuss during postnatal home visits, when it may be too late to influence practice. Timing of family counselling about newborn care practices is important to consider when designing delivery approaches. Since many of these practices need to be applied soon after birth, messages need to be given to women and families during pregnancy and reinforced after birth. Early initiation of breastfeeding did not significantly increase in intervention areas in Bangladesh, suggesting that the programme needs to examine how to improve promotion and support of breastfeeding. However, it is important to note that the rates of early breastfeeding initiation were high at baseline and there were existing nutrition interventions under the National Nutrition Program.

Home visits during pregnancy were associated with a greater likelihood of using selected practices in two countries – Malawi and Bangladesh – and a similar although non-significant trend was seen in Nepal, possibly due to inability to detect a statistically significant difference given the very small number of women reporting no home visits during pregnancy. In Bangladesh, most women received pregnancy visits, and there was a strong association between home visits and use of practices. High coverage of visits could have helped change norms, facilitating widespread uptake of these practices. Also, only home births were included in the analysis of Bangladesh data, and counselling may be more likely to change practices when a woman delivers at home.

In Malawi, home visit coverage was low and the strength of the relationship between home visits and practices is weaker. In both these countries, there may be a dose-response – while one or two home visits was associated with an increased likelihood that newborn care practices were applied, statistically significant associations were only seen when more than three antenatal home visits were received. Thus, it appears that a sufficient number of visits are needed to reinforce and support behaviours. However, it may not be feasible in all contexts for CHWs to visit homes three or more times during pregnancy, and some of the programmes were not even designed to visit women that many times. There is a need for closer examination into how programmes can deliver messages and promote behaviour change, in addition to home visits. These programmes incorporated community mobilisation and mass media messages to different extents; there is a need to understand the influence of such components and how they interact with home visits, although data were not available in these pilot areas for such analysis.

Uganda was the only country where home visits did not appear to have an association with the composite indicator. It is possible that the effect of home visits on these practices was diluted by other intervention elements, including facility quality improvement efforts and community mobilisation. The rapid changes in comparison areas suggest that other programmes and secular trends were influencing these practices, and possibly a spill-over effect from intervention areas. Practices can change rapidly and programmes come and go, so health managers need to monitor what is happening on a regular basis to ensure additional efforts to promote practices as necessary and appropriate.

In addition, it is difficult to tease out the importance of CHW characteristics on the quality of counselling and the likelihood that women and families will heed counselling advice, although they are probably important. In Malawi, where coverage of home visits was low but there was an association between home visits and practices, CHWs are trained, government-employed workers who also work at facilities, so community members recognise them as health professionals and an extension of the formal health system. That may have increased the influence of their home-based counselling on practices. In Uganda, CHWs are community members and part-time volunteers with substantially less training that may or may not be seen as part of the health system. Therefore, their home-based counselling may not be as effective in changing practices.

Although existing government structures, systems, and authorised employees or volunteers were used, implementation required additional inputs. Ministries of health in these countries are in the process of rolling out community-based newborn care programmes that were informed by the pilot programmes described here (22–25), and the amount of external implementation support varies within and across countries. Even where there is support, it has been difficult to reach every woman and newborn with home visits (26), and there are implementation challenges including the difficulty of identifying all pregnant women, ensuring and maintaining skills for large numbers of workers, and tracking programme-relevant data collected at community level without overburdening community workers (35).

Programmes such as those described here are often embedded in a long programmatic history. For example, in Nepal there was a safe delivery incentive programme to increase institutional delivery with Female Community Health Volunteers conducting pregnancy home visits that included counselling on the newborn care practices examined in this paper (36, 37). Previous efforts can have an important effect on the current programme of interest. More importantly, we should not assume that positive programme effects mean high performance continues after outside partner attention and inputs are removed. Although programme performance is often measured at the height of implementation efforts, experience demonstrates that performance often declines and most or all of the observed benefit may be lost.

Analytical limitations include differences in household survey questionnaires; variations that may affect comparability have been noted. Use of a composite indicator masks the association between home visits and each individual behaviour, some of which were not statistically significant. Questionnaires were administered to women with a live birth in the previous year and may have introduced recall or reporting bias. Research shows that women have been able to accurately recall some behaviours (particularly skin-to-skin care) but may have difficulty recalling others (38). There is the possibility that women falsely report behaviours that are believed to be socially desirable; this bias may have been stronger among women exposed to the intervention. Finally, there are many factors that contribute to newborn care practices which may not have been captured by the confounding factors in the model. Newborn care practices could have been influenced by the community engagement activities done under these and other programmes, mass media messages, other actors, and shifting social and cultural norms, which we were not able to measure and incorporate into these analyses.

Data collection efforts were not powered to measure impact on mortality. We also could not estimate the impact using modelling programmes such as the Lives Saved Tool because the effectiveness and coverage of several of the interventions included in this analysis are not currently included in the tool. Efforts to expand the Lives Saved Tool to include more of these healthy behaviours will help countries estimate mortality changes based on changes in coverage of evidence-based interventions.

## Conclusion

This analysis shows that newborn care practices can rapidly change along with the context in which they are used. Home visits by CHWs during pregnancy can play a role in improving practices in different settings. However, multiple interactions are probably needed to change behaviour, so programmes need to investigate the most appropriate and efficient ways to reach families and promote newborn care practices. The magnitude of the association between home visits and use of key practices was greatest in Bangladesh, where only non-facility births were included in the model, indicating that the impact of home visit programmes on newborn care practices may be most profound in areas with high rates of home delivery. At the same time, facility delivery appears to be the most important predictor for ensuring newborn care practices, so programmes need to take advantage of increasing facility delivery rates to ensure that all babies benefit from these practices.
